# Effects of Simvastatin Treatment on Serum Adiponectin Concentrations in Patients With Dislipidemia

**DOI:** 10.5812/ircmj.6915

**Published:** 2014-08-05

**Authors:** Atefeh Moezzi, Seyyed Mohammad Reza Parizadeh, Shima Tavallaie, Mohsen Mazidi, Fariba Afzali, Afrouz Adab, Gordon Ferns, Majid Ghayour Mobarhan

**Affiliations:** 1 Biochemistry of Nutrition Research Center, Faculty of Medicine, Mashhad University of Medical Sciences, Mashhad, IR Iran; 2 Cardiovascular Research Center, Faculty of Medicine, Mashhad University of Medical Sciences, Mashhad, IR Iran; 3 Brighton and Sussex Medical School, University of Sussex, Brighton, United Kingdom

**Keywords:** Atherosclerosis, Adiponectin, Statins

## Abstract

**Background:**

Adiponectin is an adipose tissue-derived protein with anti-inflammatory properties. Statins are a class of cholesterol-lowering drugs, widely used for treatment of cardiovascular diseases.

**Objectives:**

In the current study, we aimed to assess the effects of simvastatin on serum levels of adiponectin in patients with dyslipidemia, recruited from Ghaem Hospital, Mashhad, Iran.

**Materials and Methods:**

A total of 102 patients with dyslipidemia were treated with simvastatin or placebo during a double-blind, cross-over, placebo-controlled trial. The adiponectin levels were measured before and after each treatment period. Seventy seven participants completed the study.

**Results:**

There was a significant reduction in serum total cholesterol (approxmately 21%), low density lipoprotein-cholesterol (LDL-C) (approxmately 28%), and triglycerides (approxmately 11%), after four weeks of treatment with simvastatin (P < 0.001).

**Conclusions:**

No significant change in serum adiponectin concentrations was observed after treatment with simvastatin. This may be because of the relatively short duration of treatment and longer treatment duration may be necessary to investigation in future studies.

## 1. Background

Atherosclerosis is the major cause of cardiovascular disease (CVD) and mortality globally (1). The statin group of drugs is widely used to treat hypercholesterolaemia in patients at CVD risk (2). Statins inhibit the rate-limiting enzyme in cholesterol biosynthesis, HMG-CoA reductase, and reduce serum cholesterol levels substantially (3). Recent studies have shown that statins has have other properties in addition to lowering the cholesterol level (4), including anti-inflammatory effects (5). We recently reported a significant reduction in mean serum prooxidant-antioxidant balance (PAB) concentrations, a marker of oxidant stress after treatment with simvastatin (6). The pleiotropic effects of statins have been reported previously, but the relative importance of these effects in reducing the CVD risk has been contentious (7). Adiponectin is a hormone secreted by adipocytes with antidiabetic, antiatherogenic, and anti-inflammatory properties (8). Recent studies have demonstrated that adiponectin exerts its effects through two receptors, known as AdipoR1 and AdipoR2 (9). Saito et al. recently demonstrated that pravastatin abrogated the decrease in AdipoR1 expression in myocardial tissue, independently of changes in serum cholesterol and insulin levels (10). Several other studies have explored the biological roles of adiponectin, which make it a potential therapeutic target. The effects of statins on myocardial expression of adipoR1 and serum levels of adiponectin may be important properties for their application in treatment of ischemic heart disease and heart failure (11).

## 2. Objectives

Because of the potential importance of the adiponectin levels in CVD, we investigated the effects of simvastatin on serum levels of adiponectin in a group of patients with established atherosclerosis or at risk for CVD.

## 3. Materials and Methods

A total of 102 male and female patients, aged 20–88 years old, who were not originally taking lipid-lowering agents, were recruited from the lipid clinics of Ghaem Hospital, a teaching hospital located in Mashhad, Iran, between June 2010 and August 2012. In addition to a history of not taking statins, other inclusion criteria were any of the following conditions (based on the NCEP-ATP III (National Cholesterol Education Program) guidelines (12):

1. patients with < 2 risk factors (except diabetes mellitus) for coronary heart disease (CHD) and 160 mg/dL < low-density lipoprotein cholesterol (LDLc) < 190 mg/dL, or,
2. patients with ≥ 2 risk factors (except diabetes mellitus) for CHD and 130 mg/dL < LDLc < 160 mg/dL.

The CVD risk factors were defined as age > 65 years old, hypertension (defined as taking any antihypertensive medication, or systolic blood pressure of > 140 mmHg or diastolic blood pressure of > 90 mmHg), diabetes mellitus (defined as fasting blood sugar (FBS) ≥ 126 mg/dL), positive family history of CVD, smoking, male sex, and obesity [defined as body mass index (BMI) ≥ 30 kg/m^2^]. The exclusion criteria were a history of malignancy, recent history of infections, connective tissue disorders, treatment with immunomodulatory drugs (e.g. corticosteroids), liver or renal disease, leukocytosis (white blood cell count > 10000/L), thrombocytosis (platelet count > 450,000 × 10^9^/L), and anemia (hematocrit < 40%). Each subject signed an informed written consent to participate in the study, which had previously been approved by the Ethics Committee of Mashhad University of Medical Sciences (date: Feb 12, 2010; code: 88585).

### 3.1. Study Design

The study was a randomized placebo controlled cross-over trial, in which each patient received simvastatin or a placebo and then crossed over to the alternate regimen. Each treatment period was 30 days and there was a two-week washout interval between the treatment periods. The doses of all other medications remained unchanged during the experimental period and the patients were advised not to change their lifestyles during the study. At the first visit, patients were randomly assigned (random number tables) to one of the two treatment regimens. Fifty one patients were provided with simvastatin 40 mg/day for 30 days and the other 51 received a placebo (simply prepared by filling empty capsules which were matched for size and color with simvastatin capsules and contained starch instead of simvastatin) for 30 days. After a further two-week wash-out period, the patients crossed over to the other form of treatment. The sample size was based on the between-group mean comparison formula, according to the study conducted by Hu et al. with confidence interval of 95%, and power of 80% was calculated as for at least 45 subjects for each arm (13).

### 3.2. Anthropometric Measurements

Anthropometric parameters including weight, height, BMI, waist and arm circumferences, and body fat were measured using standard procedures, as described previously (14, 15).

### 3.3. Blood Sampling

Blood samples were collected at four time points for each subject (before and after starting each period). Blood samples for laboratory assays were obtained on the day of sampling after 12 hours of fasting. Following venipuncture, the blood samples were collected into vacutainer tubes and centrifuged at 10000 g for 15 minutes at 4°C. After separation, aliquots of serum were frozen at -80°C until analysis.

### 3.4. Biochemical Analysis

A full fasted lipid profile comprising total cholesterol, triglycerides, high-density lipoprotein cholesterol (HDLc) and LDLc were determined for each subject. Serum lipid and fasting blood glucose (FBG) concentrations were measured enzymatically using commercial kits.

### 3.5. Chemicals

Serum levels of adiponectin were measured in duplicates, using an enzyme-linked immunosorbent assay kit.

### 3.6. Statistical Analysis

Values were expressed as means ± SD, or in the case of non-normally distributed data, as median and inter-quartile range. The comparison between pre- and post-treatments was performed using t-test. The mixed-model analysis of variance for 2 × 2 cross-over studies was fitted and the analyses were performed using the statistical analysis software (SAS version 8). The differences of these cross-over analyses were compared; they were normally distributed. As an indicator of the therapeutic benefit magnitude, the effective levels of changes in adiponectin concentration between the placebo and simvastatin groups were calculated using the Cohen's d formula: the absolute difference between the placebo and simvastatin groups/standard deviation of difference. A two-sided P value of < 0.05 was considered statistically significant.

## 4. Results

Of the 102 subjects who entered the study, 25 (24.5%) did not complete it, leading to a final sample size of 77 (78.2%). The reasons for drop-outs were noncompliance with the study protocol (n = 21), drug intolerance (n = 2) and moving to another city (n = 2). To eliminate the possibility of a carry-over effect from one treatment period to another, the baseline values of biochemical and anthropometric parameters before the first treatment period were compared with those before the second treatment period. No significant difference was found in this analysis (P > 0.05). In addition, age, sex, hyperlipidemia, BMI, presence of hypertension, diabetes, and smoking status were not significantly different between the two groups (Table 1).

### 4.1. Effects of Administration of Simvastatin Versus Placebo on Weight, Body Mass Index and Fasting Blood Sugar

Neither simvastatin nor the placebo affected the FBG level significantly (P > 0.05). The mean baseline values for BMI and weight, however, were significantly different between the first and second periods of treatment (P < 0.05) (Table 1).

**Table 1.** Comparison of Baseline Characteristics of Subjects ^a,b,c^

Parameters	Statin-Placebo	Placebo-Statin	P Value
**Age, y**	46.00 ± 14.83	44.18 ± 12.07	> 0.05
**Female sex, %**	36	26	> 0.05
**BMI, kg/m** ^**2**^	31.12 ± 6.43	28.83 ± 6.18	> 0.05
**SBP, mmHg**	111 ± 16	109 ± 15	> 0.05
**DBP, mmHg**	66 ± 12	64 ± 13	> 0.05
**Smoking, %**	2	4	> 0.05
**Diabetics, %**	9	3	> 0.05
**Hyperlipidemia, %**	26	26	> 0.05
**Hypertension, %**	2	6	> 0.05

^a^ Abbreviations: BMI, body mass index; SBP, systolic blood pressure; DBP, diastolic blood pressure.

^b^ The between-group comparisons were assessed using independent-samples t-test for normally distributed data, Mann–Whitney U-test for nonparametric data, and χ2 test for categorical data.

^c^ Data are presented as mean ± SD.

### 4.2. Effects of Administration of Simvastatin Versus Placebo on Lipid Profile

As expected, lipid parameters reduced significantly after four weeks of treatment with simvastatin (P < 0.001); total cholesterol (~21%), LDLc (~28%), and triglyceride (~11%). The corresponding values for placebo were ~0.9% decrease in total cholesterol, ~1% decrease in LDLc, and ~3% increase in triglycerides. HDLc did not change significantly with either of the treatments (P > 0.05) (Table 1).

### 4.3. Effect of Administration of Simvastatin Versus Placebo on Adiponectin Values

Statin therapy did not have a significant effect on serum levels of adiponectin in either the statin-placebo or the placebo-statin group (P > 0.05, Table 2). The intra-assay coefficient of variations (CV) of adiponectin was 3.5% while the inter-assay CV was 4.4%.

**Table 2.** Changes in Biochemical and Anthropometric Data in the Simvastatin- and Placebo-Treated Groups ^a,b,c^

Study Groups (n = 51)	First Pretreatment	Post-treatment Period	Second Pretreatment	Post-treatment Period	Period effect	Treatment effect (S vs. P)
**FBG, mg/dL**					(P > 0.05)	(P > 0.05)
Statin-placebo	86.46 ± 19.52	85.58 ± 17.12	81.28 ± 15.83	81.09 ± 13.65		
Placebo-statin	104.36 ± 47.61	101.71 ± 45.86	103.5 ± 45.55	99.10 ± 33.28		
**Weight, kg**					(P < 0.01)	(P > 0.05)
Statin-placebo	71.59 ± 22.02	72.42 ± 19.24	73.80 ± 18.60	75.27 ± 18.55		
Placebo-statin	78.04 ± 16.63	77.17 ± 16.30	76.99 ± 15.50	76.71 ± 16.41		
**BMI, kg/m** ^**2**^					(P < 0.05)	(P > 0.05)
Statin-placebo	28.83 ± 6.18	28.30 ± 6.19	28.95 ± 5.90	29.09 ± 5.91		
Placebo-statin	31.12 ± 6.41	30.83 ± 6.36	30.33 ± 7.41	31.25 ± 6.04		
**TC, mg/dL**					(P > 0.05)	(P < 0.001)
Statin-placebo	203.02 ± 36.11	152.48 ± 41.60	181.69 ± 30.65	182.38 ± 37.64		
Placebo-statin	193.32 ± 39.65	191.06 ± 38.04	194.85 ± 37.75	160.37 ± 60.81		
**LDLc, mg/dL**					(P > 0.05)	(P < 0.001)
Statin-placebo	131.44 ± 28.46	87.35 ± 35.01	119.75 ± 26.44	121.09 ± 23.86		
Placebo-statin	118.38 ± 30.48	115.22 ± 35.03	121.75 ± 28.25	92.50 ± 46.48		
**HDLc, mg/dL**					(P > 0.05)	(P > 0.05)
Statin-placebo	44.08 ± 10.80	43.31 ± 12.19	40.36 ± 13.34	41.79 ± 14.97		
Placebo-statin	42.40 ± 11.92	42.64 ± 13.32	44.55 ± 12.42	45.96 ± 14.58		
**TG, mg/dL**					(P > 0.05)	(P < 0.001)
Statin-placebo	1.325 (1.00, 2.0)	1.25 (91.5, 2.2)	1.225 (87.5, 1.90)	1.05 (80.25, 1.56)		
Placebo-statin	1.24 (93.00, 1.73)	1.12 (73.0, 1.5)	1.250 (82.0, 1.77)	1.14 (90.00, 1.53)		
**APN, ng/mL**					(P > 0.05)	(P > 0.05)
Statin-placebo	0.56 ± 0.29	0.63 ± 0.29	0.73± 0.32	0.59 ± 0.28		
Placebo-statin	0.55± 0.33	0.63 ± 0.32	0.63 ± 0.32	0.56 ± 0.27		

^a^ Abbreviations: BMI body mass index; FBS, fasting blood sugar; TC, total cholesterol; HDL, high-density lipoprotein; LDL, low-density lipoprotein; TG, triglycerides; APN, adiponectin.

^b^ Values are expressed as means ± SD for normally distributed data and median and interquartile range. Statin-placebo group took statin at first, while placebo-statin group received statin following placebo.

^c^ Defined as comparison of mean values between the first and second periods.

## 5. Discussion

In the current study, we found that simvastatin did not significantly affect the serum adiponectin concentrations after four weeks of treatment. However as expected, total cholesterol, LDLc and triglyceride levels reduced significantly after four weeks of treatment with simvastatin (P < 0.001).

### 5.1. Statins and Adiponectin

Epidemiological studies have demonstrated an inverse correlation between adiponectin and the following parameters: BMI, type 2 diabetes mellitus, insulin resistance, hyperlipidemia, triglyceride level, and high blood pressure (16). Most experimental studies have emphasized on the relationship between increasing adiponectin levels and cardiovascular protective properties. Serum adiponectin concentrations have been reported to increase following treatment with several drugs, including statins. Recent findings have demonstrated the positive effects of simvastatin, pravastatin, rosuvastatin and atorvastatin on adiponectin values (13, 17-20).

### 5.2. Simvastatin and Adiponectin

Statin treatment is associated with a number of pleiotropic effects, including a reduction in serum markers of inflammation (21). Simvastatin is widely used, relatively potent, and lipophilic (22); thus, we used simvastatin in this study to investigate its effects on serum adiponectin. Hu et al. recently evaluated the effects of simvastatin on adipokines. A significant increase in serum adiponectin levels was observed after three months of simvastatin treatment. Longer treatment period (three months versus four weeks study) was one of the differences between the current study and the previous study (17). Another difference was the acute weight loss of patients during this study.

### 5.3. Study Limitations

The present study had several limitations. First, 25 subjects did not complete the study due to noncompliance of the study protocol or drug intolerance. Second, simvastatin was administered at a dose of 40 mg/day for a limited period (30 days). Other long-term studies observed positive effects of statin therapy on serum adiponectin concentration, suggesting that long-term studies may be necessary to demonstrate the effects of simvastatin on adiponectin (17). Moreover, the short-term changes of weight were clearly a potential confounding factor which might have affected the results. However, the weight loss difference between the two groups was not significant. We did not find a significant change in serum adiponectin levels after treatment with 40 mg/day of simvastatin. This might be due to the short duration of treatment. Some differentiating factors of this study were being randomized, double-blinded, placebo-controlled, and cross-over designed.

The main strength of the present study was being based on a robust placebo-controlled and cross-over design as well as being conducted in a target population, not under concomitant lipid-lowering therapy. Therefore, many of the confounding factors that may generally affect lipid alterations were eliminated from the present trial. A number of limitations need to be acknowledged for the present trial: First, the small number of participants and second the short treatment duration; a long-term cross-over trial may be necessary. However, further attempts must be undertaken to investigate other mechanisms of action for statins other than their lipid lowering property, to develop their therapeutic roles in treatment of cardiovascular diseases.
